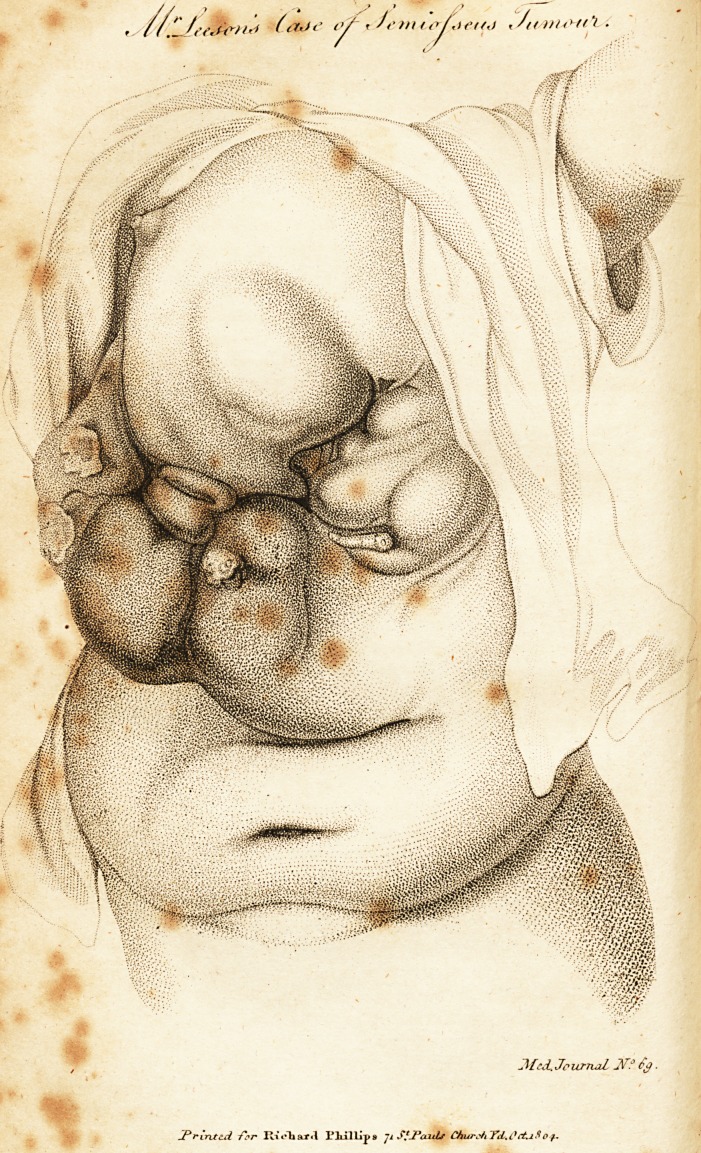# Mr. Leeson's Case of Semiosseus Tumour

**Published:** 1804-11-01

**Authors:** B. Leeson

**Affiliations:** Grantham, Lincolnshire


Mr. Leeson's Case of Semiosseus Tumour.

### *To the Editors of the Medical and Physical Journal.*

Gentlemen,

In your very useful publication for August, 1803, you favoured me with the insertion of the case of Francis Otter; on which I proposed to furnish some pathological observations in a future number. Different circumstances prevented the completion of this design for a considerable time, during which the external appearance of the tumour has become so much changed, that the reasonings, formerly made, on the cause and origin of the disease, might now perhaps be scarcely considered to apply. I therefore laid aside my intention, until a more minute inquiry might confirm or destroy the opinions I had formed on the subject; which examination, the daily expected death of the patient promised to allow. But being, by the friendly assistance of Mr. Hartley, furnished with a drawing of the tumour in the present state, I have enclosed it, together with a short account of the progress of the disease during the the twelve months which have elapsed since my last communication. At the date of the former account, July, 1803, it was observed, that the tumour was very much reduced, though some discharge continued; that this was probably only kept up by exfoliations about to take place, at the evacuation of which, it was to be expected that the sores would heal. This expectation was supported by the frequent escape of small pieces of bone, some in a boney, some in a cartilaginous state. After some time, small cysts arose on the surface occupied by the tumour, some of which, on being opened, discharged a gelatinous fluid, not uncommonly mixed with a half solid substance, appearing formerly to have been bone. These cysts continued to multiply, and when left to themselves ulcerated with frequent and considerable hæmorrhage, leaving unpleasant and painful sores. Still the patient maintained his strength, his appetite being unimpaired.

The annexed drawing may furnish some idea of the present appearance of the parts, and if compared with the engraving published in your Journal for August, 1803, will show, that the space now occupied by the various cysts is little less than that formerly filled up by the uniform tumour. The cysts, when examined by a probe, are found to communicate with each other; but the frequent occurrence of hæmorrhage has of late forbidden any very accurate investigation. On the right side of the drawing will be seen two sores, remaining from cysts lately ruptured; the centre shews a deep sulcus, from whence there is a constant discharge of ichorous matter, and not uncommonly of blood: below are small mammary projections about to be the outlets to the cysts beneath them.

Having stated thus much in explanation of the drawing, allow me to call your attention to the origin, and thence to the probable cause of the tumour; in the progress of which, I conceive, nature has exhibited some of her most wonderful exertions. By reference to the first communication on this subject, it will be found, that the original injury was the fracture of a rib in a subject very far advanced in years. The accident was neglected, and an irregular callus was formed; a small tumour was observed to follow the immediate infliction of the injury. This tumour, I suspect was occasioned by a partial division of the intercostal artery, which pouring out blood at the time the process of adhesion and consolidation of the rib was going on, formed to itself a cyst. This cyst was for a short time stationary; but blood constantly flowing into it, produced an increase of bulk, from whence pressure on the surrounding parts was produced, and the consequence of pressure was was absorption. These several actions continued until the integuments were extended so much beyond the usual bounds, as to occasion the tumour first described, and ultimately to give way. Since that event, the irritation of different detached portions of ossific matter, and the effort to expel them, has occasioned the present tubercular appearance of the surface. Is it not a striking illustration of one of the laws of Nature, that in the progress of this tumour, all the effort for evacuation should be externally, and not through the peritoneum?

I am, &c.

*Grantham, Lincolnshire, Sept.* 13, 1804. B. LEESON, Jun.

## Figures and Tables

**Figure f1:**